# Supplementary material for: Overexpression Bombyx mori HEXIM1 Facilitates Immune Escape of Bombyx mori Nucleopolyhedrovirus by Suppressing BmRelish-Driven Immune Responses
Source: Viruses. 2022 Nov 25;14(12):2636. doi: 10.3390/v14122636 (PMC9782744; doi:10.3390/v14122636)
Supplement: Supplementary file 1 [file viruses-14-02636-s001.zip › Supplements/Supplementary Figure legend.pdf]

Figure S1. Structural domain analysis and amino acid sequence analysis of HEXIM1. (A, B) Multiple amino acid sequence alignment of HEXIM1 (A) and structural domain analysis of HEXIM1 (B). Species included Bombyx mori, Drosophila melanogaster-A, Dro-sophila melanogaster-B, Mus musculus and Homo sapiens.

Figure S2. The phylogenetic tree of HEXIM1. The phylogenetic tree of HEXIM1 from Lepidoptera, Diptera and Hymenoptera was built by neighbor-joining method in MEGA 6 with a bootstrap value of 1000.

Figure S3. BmHEXIM1 and PK1 or viral RNA polymerase colocalized intracellularly.

(A) BmHEXIM1 and PK1 or two subunits of viral RNA polymerase, P47 and LEF-9 were co-expressiond visualized by immunofluorescence microscopy in BmN cells. Nuclei were visualized by counterstaining with DAPI. Representative photomicrographs show that BmHEXIM1–Red was localized at the edge of the cell and some throughout the cytoplasm and VS region. PK1, P47 and LEF-9–Green was primarily localized at the cytoplasmic membrane along with some in the cytoplasm.

(B) Immunoprecipitations of lysates from BmN cells transfected with flag tagged BmHEXIM1 together with infected by recombinant virus overexpressed HA tagged PK1, P47 and LEF-9 using anti-HA/ anti-FLAG antibodies were performed at 48 h. Input and precipitate samples were subjected to SDS-PAGE and Western blot, and BnHEXIN1, PK1, P47 and LEF-9 were detected using anti-FLAG and anti-HA antibodies, respectively.
