# Supplementary material for: Overexpression Bombyx mori HEXIM1 Facilitates Immune Escape of Bombyx mori Nucleopolyhedrovirus by Suppressing BmRelish-Driven Immune Responses
Source: Viruses. 2022 Nov 25;14(12):2636. doi: 10.3390/v14122636 (PMC9782744; doi:10.3390/v14122636)
Supplement: Supplementary file 1 [file viruses-14-02636-s001.zip › Supplements/Supplementary Table S1.pdf]

Supplementary Table S1. Primers used in this study.

Primer name	Primer sequences
qPCR-RpL32-F	CAGGCGGTTCAAGGGTCAATAC
qPCR-RpL32-R	TGCTGGGCTCTTTCCACGA
qPCR-HEXIM1- F	TAGAAGATGGCGAACCGACG
qPCR-HEXIM1 -R	GAACGGGGCATGAGTTTTCG
qPCR-SPT5-F	GGGCGCTGAACCAGAAGAAT
qPCR-SPT5-R	TGTTCACCGGTGCCTTTGAT
qPCR-CDK9-F	GTCGCAAAGTCTGGACAAGC
qPCR-CDK9-R	CCATGATACATCCGGCTCCC
qPCR-NELF-D-F	TGTACGATGCACCGGAAACT
qPCR-NELF-D-R	TCACCACTTTCGCTATTCGGT
qPCR-VP39-F	ATTGATCGCCAACACCACCT
qPCR-VP39-R	AGACACCACAAACCCGAACA
qPCR-POLH-F	GGTGCTACAAGTTCCTCGCT
qPCR-POLH-R	GTTGGTGTACTCGCTGTGGA
qPCR-P10-F	AACGGGCTGGAAGAATCGTT
qPCR-P10-R	GAGCAGTGTCACCGGTCAAT
qPCR-Lef8-F	CCACAAGCTTGCCCTTCAAC
qPCR-Lef8-R	AAATTGTGGACGTTGGTGCG
qPCR-Lef4-F	TTCCACGATGGCGAACAAGA
qPCR-Lef4-R	TATTCGAGACGTATGCGGGC
qPCR-Lef9-F	TCGCCAGTTCAAACGCTAGT
qPCR-Lef9-R	TGTCTACAAATTGGCCGCCT
qPCR-P47-F	GTCATGTCCGTGAAACGTGC
qPCR-P47-R	AACGGGTTCGATTACACGCT
qPCR-KMO-F	GTTCCTTGGTACCGTCACGA
qPCR-KMO-R	AGTTCGCTTAAATAACGACACAA
qPCR-BCAT-F	GAACGAGCGAGGAAGTGACA

qPCR-BCAT-R	CAACGGAAGCGAGCACAAAA
qPCR-CecA-F	TCGCTTGCCCTATGACG
qPCR-CecA-R	TGAGCCCAGGTGGAAACT
qPCR-CecB-F	CCTATCCTTCGTCTTCGCTCT
qPCR-CecB-R	TAGCTTTAGCCGAACCAAGG
qPCR-Relish-F	GTCGTTGTTCCGGGCGTCC
qPCR-Relish-R	TCCACACGCGGTGGCCATTC
qPCR-STING-F	AGCCGTCAACCGTCACTT
qPCR-STING-R	GAAAACCTACGAATCTGT
