# Supplementary figures and images for: Overexpression Bombyx mori HEXIM1 Facilitates Immune Escape of Bombyx mori Nucleopolyhedrovirus by Suppressing BmRelish-Driven Immune Responses
Source: Viruses. 2022 Nov 25;14(12):2636. doi: 10.3390/v14122636 (PMC9782744; doi:10.3390/v14122636)

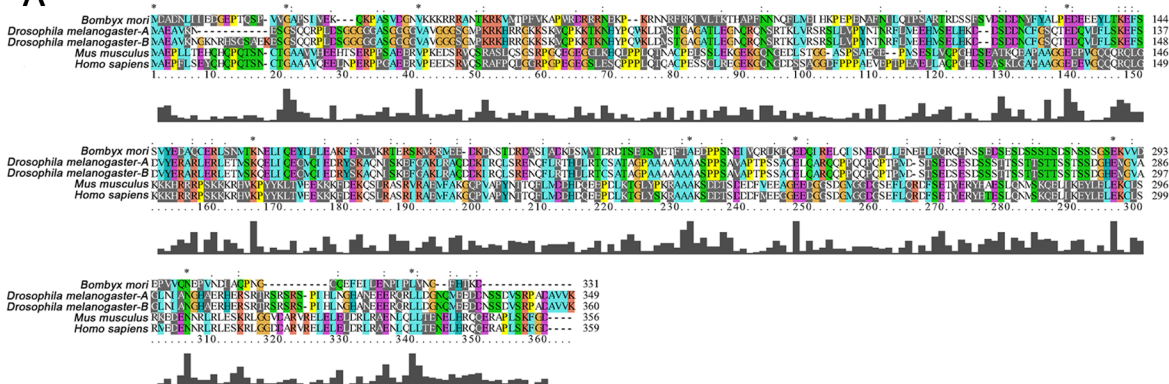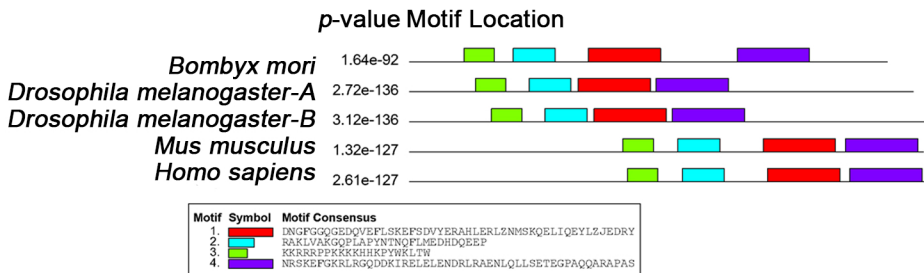

Supplement: Supplementary file 1 [file viruses-14-02636-s001.zip › Supplements/Figure S1.pdf]

Tree scale: 0.1

Colored ranges

- Lepidoptera
- Diptera
- Hymenoptera

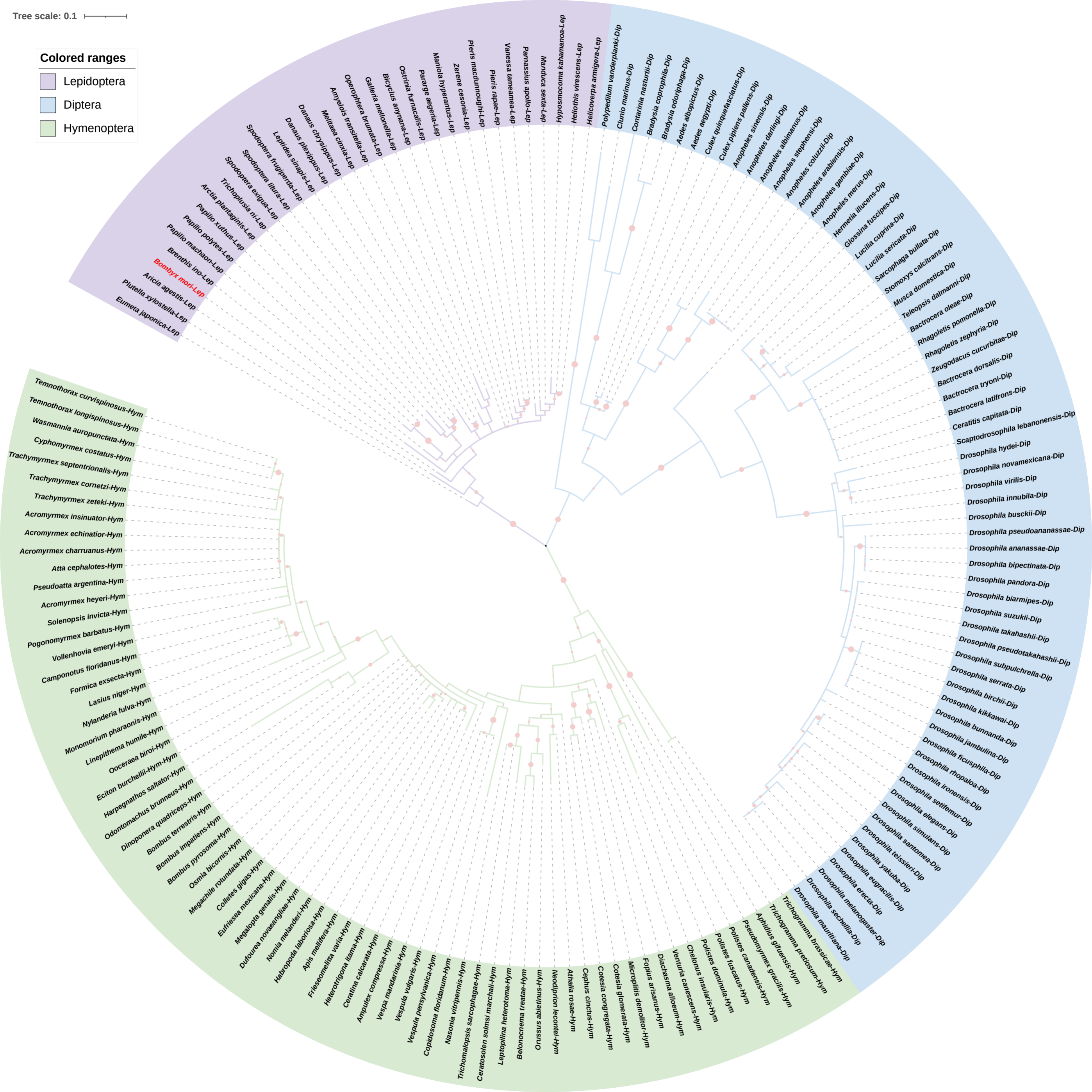

Supplement: Supplementary file 1 [file viruses-14-02636-s001.zip › Supplements/Figure S2.pdf]

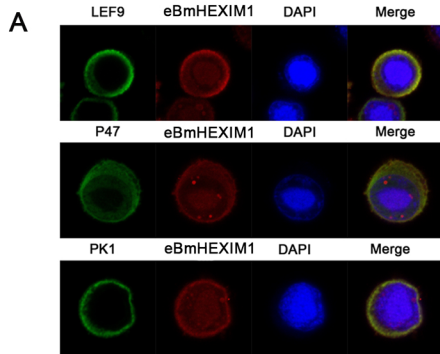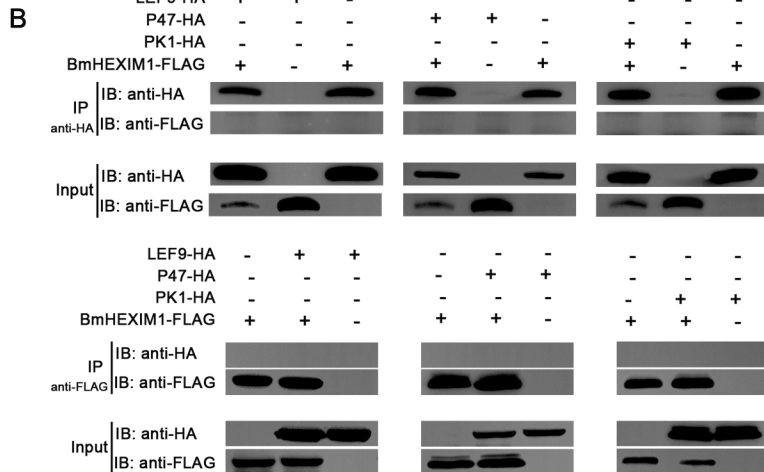

Supplement: Supplementary file 1 [file viruses-14-02636-s001.zip › Supplements/Figure S3.pdf]
